# Short-term results of the Warden technique: a retrospective single-center study

**DOI:** 10.1186/1749-8090-8-S1-P95

**Published:** 2013-09-11

**Authors:** G Liguori, T Camargo, D Akerman, J Penha, L Miana, L Caneo, C Tanamati, M Jatene

**Affiliations:** 1 Heart Institute (InCor), University of Sao Paulo Medical School, Sao Paulo, Brazil

## Background

The Warden technique was introduced in an attempt to decrease the incidence of sinus node dysfunction and venous obstruction after the repair of partial anomalous pulmonary venous connection to the superior vena cava. In this study, we sought to report and evaluate the immediate outcomes of our experience with the Warden technique.

## Methods

We conducted a retrospective study of 9 patients with anomalous drainage of the pulmonary veins to superior vena cava who have undergone the Warden technique during the year of 2011. We analyzed medical records, surgical reports and results of complementary tests.

## Results

Besides the anomalous pulmonary venous connection to the superior vena cava, 5 (56%) patients also presented an ostium secundum or patent foramen ovale atrial defect and 2 (22%) presented persistent left superior vena cava. One patient presented Turner Syndrome. Before surgery, 3 (33%) patients presented some form of conduction disorder: two right bundle branch block and one atrial extrasystole. The mean age at surgery was 10.8±7.6 (min=2, max=29.7) years, and the distribution of the sexes was 2:1, the majority being male. None patient underwent reoperation or evolved to death during the follow-up period. After surgical repair, a forth patient presented with right bundle branch block. The degree of right chambers dilatation improved significantly at both atrial (p=0,002) and ventricular (p=0,046) level, after surgical repair. Three (33%) patients had postoperative tricuspid and/or pulmonary insufficiency. No venous obstruction occured.

## Conclusion

Short-term results with the Warden technique were satisfactory. Althought postoperative valvopathies and arrhythmias seems to be a risk, longer follow-up is required to evaluate its impact on morbidity and mortality.

